# Editorial: Nutrition for sustainable weight management post-bariatric surgery

**DOI:** 10.3389/fnut.2026.1816785

**Published:** 2026-04-14

**Authors:** Bart Torensma, Mohamed Hany, Edo Aarts, Frits Berends

**Affiliations:** 1 Department of Clinical Epidemiology, Erasmus University Rotterdam, Rotterdam, Netherlands; 2 WeightWorks Clinics, Amersfoort, Netherlands; 3 Medical Research Institute, Alexandria University, Alexandria, Egypt

**Keywords:** amino acid metabolism, anemia, bariatric metabolic surgery, nutritional deficiency, post-operative outcomes, precision medicine, weight management, weight recidivism

The global obesity epidemic continues to increase, now affecting over one billion individuals worldwide (1) and placing a heavy burden on healthcare systems, economies, and individual quality of life (2). Metabolic Bariatric Surgery (MBS) has proven to be the most effective long-term intervention for severe obesity, delivering sustainable weight loss and amelioration of obesity-related associated medical problems that remain largely unattainable through conservative measures alone (3–5). Yet, the narrative surrounding MBS has matured considerably. Despite its demonstrated efficacy in achieving significant weight reduction, maintaining long-term weight loss remains a substantial challenge for many patients (6). This challenge is intricately linked to post-surgical dietary habits, nutritional intake, and the complex interplay of behavioral, psychological, and socioeconomic factors that shape the post-operative trajectory. Weight regain, nutritional deficiencies, and unanticipated complications such as alopecia and behavioral changes are increasingly documented (3, 7, 8), underscoring the need for comprehensive, individualized approaches to post-bariatric care.

This Research Topic, *Nutrition for sustainable weight management post-bariatric surgery*, was conceived to explore innovative, evidence-based nutritional strategies to bolster long-term weight management for post-MBS patients. Its scope encompasses a multifaceted approach: identifying optimal dietary patterns and the role of micronutrient supplementation, integrating behavioral health interventions to support dietary adherence, and investigating the psychological and social determinants of eating behaviors after surgery. The underlying premise is that long-term success hinges not only on what patients eat but also on their individual relationships with food, their genetic and metabolic profiles, and the quality of multidisciplinary support they receive.

By fostering dialogue across disciplines, this Research Topic seeks to generate novel insights and practical evidence that healthcare professionals can use to support their patients throughout the continuum of post-bariatric care. The four contributions assembled here span the spectrum from broad narrative reviews to focused prospective cohort studies, collectively addressing how to optimize nutritional and clinical outcomes after MBS—not merely in the weeks following the procedure, but over the long term.

The challenge of recurrent weight gain remains one of the most clinically significant concerns in bariatric medicine, and it is fitting that this Research Topic opens with a comprehensive perspective on the topic. Xiao et al. provide a narrative review addressing the prognostication of post-bariatric surgery outcomes and the management of recurrent weight gain and diabetes recurrence. Their work synthesizes the evidence on predictive factors—including pre-operative BMI, dietary habits, physical activity levels, and mental health status—that influence individual trajectories after surgery. Importantly, the authors emphasize that proactive interventions targeting both pre- and post-operative eating behaviors and lifestyle adjustments are more important than investigating pre-operative risk factors alone. The review further discusses the growing role of pharmacological agents, particularly GLP-1 receptor agonists such as semaglutide and liraglutide (9), as well as endoscopic and revisional surgical interventions for patients who experience suboptimal outcomes. By integrating dietary pattern optimization, pharmacotherapy, and procedural revision into a single framework, Xiao et al. make a compelling case that a multimodal, longitudinal approach is essential for managing the complex phenomenon of recurrent weight gain— a message that resonates strongly with the broader aims of this Research Topic.

From the broader landscape of weight management, this Research Topic moves to a more focused yet equally critical nutritional concern. Nutritional deficiencies following MBS are well-documented, yet certain pre-operative risk factors for these deficiencies remain insufficiently addressed in clinical practice. Nie et al. present a multicenter cohort study examining the impact of pre-operative anemia on post-operative anemia and related nutritional abnormalities. Among 452 patients followed for1 year after bariatric surgery, 11.7% presented with pre-operative anemia. Their findings are striking: patients with pre-operative anemia were significantly more likely to develop post-operative anemia (OR 3.52, 95% CI 1.83–7.06), ferritin deficiency (OR 3.77, 95% CI 1.74–8.17), and low transferrin saturation levels (OR 4.12, 95% CI 2.16–7.84). These associations persisted after full covariate adjustment, demonstrating a robust and independent relationship. Current clinical guidelines do not routinely recommend the management of anemia prior to bariatric surgery (3), yet these data make a compelling case for active pre-operative screening and correction of iron status. This study highlights a potentially modifiable risk factor that is frequently overlooked in pre-operative optimization protocols, and it speaks directly to the Research Topic's emphasis on supplementation strategies and the prevention of nutritional deficiencies.

An underappreciated but distressing complication of bariatric surgery is post-operative alopecia, which can significantly impact patient wellbeing, body image, and overall satisfaction with surgical outcomes. Wang et al. contribute a prospective cohort study examining the correlation between changes in the serum amino acid spectrum and alopecia in patients with obesity undergoing laparoscopic sleeve gastrectomy (LSG). Among 67 patients, the authors demonstrate that LSG significantly alters amino acid profiles, with notable decreases in arginine, alanine, threonine, branched-chain amino acids (valine, isoleucine, leucine), and aromatic amino acids (tyrosine, phenylalanine, tryptophan), while serine and glycine increased. Crucially, serum leucine concentration at 3 months post-operatively was identified as an independent risk factor for moderate-to-severe alopecia (OR 1.119, 95% CI 1.006–1.245). This work opens a novel window into the metabolic consequences of bariatric surgery at the amino acid level and suggests that targeted nutritional monitoring could help predict and potentially prevent this common yet often neglected complication. The findings contribute to the growing body of evidence that post-MBS nutritional care must extend beyond traditional micronutrient surveillance to encompass the broader metabolic landscape, including protein and amino acid homeostasis.

The final contribution broadens the perspective beyond classical nutrition to encompass the genetic and psychosocial dimensions of MBS outcomes. Hanna et al. present a narrative review exploring how precision medicine approaches—incorporating genetic polymorphisms, neurotransmitter signaling pathways (particularly catecholamine and dopaminergic systems), and psychosocial risk profiling—can be leveraged to improve outcome prediction and personalize post-operative care. The authors draw particular attention to the phenomenon of addiction transfer, whereby patients who have undergone bariatric surgery may develop new substance or behavioral addictions during recovery, a concern rooted in shared neurobiological reward pathways and reward deficiency syndrome. This paper underscores a fundamental premise of this Research Topic: that sustainable weight management after bariatric surgery requires not only nutritional optimization but also careful attention to the neuropsychiatric and behavioral architecture of individual patients. By detailing genetic markers and psychosocial screening tools that may predict susceptibility to weight regain and maladaptive behaviors, Hanna et al. lay the groundwork for a truly personalized approach to post-MBS care—one in which treatment plans are tailored to the biological and psychological profile of each patient.

Taken together, the four contributions in this Research Topic paint a nuanced and clinically meaningful picture of the post-bariatric landscape. Several overarching themes emerge. First, the pre-operative period is not merely a waiting room for surgery but a critical window for optimization—whether that involves correcting anemia (Nie et al.), assessing psychosocial risk and genetic predisposition (Hanna et al.), or establishing baseline dietary and lifestyle patterns (Xiao et al.). Second, the post-operative phase demands sustained, multidimensional surveillance that extends well beyond weight monitoring to include micronutrient status, amino acid metabolism, and behavioral health. Third, the field is moving toward precision and individualization: from one-size-fits-all supplementation protocols toward targeted interventions informed by genetic, metabolic, and psychosocial profiling. These themes collectively reflect the multifaceted vision upon which this Research Topic was founded.

These studies also highlight important gaps that warrant further investigation. The amino acid–alopecia relationship identified by Wang et al. warrants validation in larger, multi-ethnic cohorts and exploration of the underlying mechanistic pathways, including potential interactions with iron and zinc metabolism. The pre-operative anemia findings of Nie et al. call for prospective interventional trials examining whether systematic correction of anemia before surgery translates into measurably improved post-operative nutritional outcomes. The precision medicine framework proposed by Hanna et al. requires operationalization—developing practical, cost-effective clinical tools that can integrate genetic and psychosocial data into routine pre-operative assessment algorithms. And the comprehensive overview by Xiao et al. reinforces the urgent need for standardized long-term follow-up protocols that capture the full spectrum of post-bariatric outcomes, from metabolic and nutritional parameters to psychological wellbeing and quality of life. Moreover, several topics originally envisioned for this Research Topic—including the role of gut microbiota in post-surgical nutritional absorption, the impact of socioeconomic and cultural factors on dietary adherence, and the synergistic effects of combined dietary and physical activity programs—remain fertile ground for future research and warrant dedicated investigation.

In conclusion, this Research Topic reaffirms that MBS is not an endpoint but a beginning. The evidence assembled here argues persuasively for a paradigm shift: from a surgical-centric model to an integrated, patient-centered framework in which nutrition, metabolism, genetics, and psychosocial factors are woven into a continuous thread of care. As MBS rates continue to rise globally, the imperative to develop and implement holistic, evidence-based post-operative management strategies has never been greater. It is our hope that the insights gathered in this Research Topic will stimulate further research, inform clinical practice, and ultimately contribute to more durable and holistic outcomes for patients navigating life after bariatric surgery.

## References

[1] NCD Risk Factor Collaboration (NCD-RisC). Worldwide trends in underweight and obesity from 1990 to 2022: a pooled analysis of 3663 population-representative studies with 222 million children, adolescents, and adults. Lancet. (2024) 403:1027–50. doi: 10.1016/S0140-6736(23)02750-2 38432237 PMC7615769

[2] Nagi MA Ahmed H Rezq MAA Sangroongruangsri S Chaikledkaew U Almalki Z . Economic costs of obesity: a systematic review. Int J Obes. (2024) 48:33–43. doi: 10.1038/s41366-023-01398-y 37884664

[3] Eisenberg D Shikora SA Aarts E Aminian A Angrisani L Cohen RV . 2022 American Society for Metabolic and Bariatric Surgery (ASMBS) and International Federation for the Surgery of Obesity and Metabolic Disorders (IFSO): indications for metabolic and bariatric surgery. Surg Obes Relat Dis. (2022) 18:1345–56. doi: 10.1016/j.soard.2022.08.013 36280539

[4] Pipek LZ Moraes WAF Nobetani RM Cortez VS Condi AS Taba JV . Surgery is associated with better long-term outcomes than pharmacological treatment for obesity: a systematic review and meta-analysis. Sci Rep. (2024) 14:9521. doi: 10.1038/s41598-024-57724-5 38664450 PMC11045962

[5] Courcoulas AP Johnson E Arterburn DE Haneuse S Herrinton LJ Fisher DP . Reduction in long-term mortality after sleeve gastrectomy and gastric bypass compared to nonsurgical patients with severe obesity. Ann Surg. (2023) 277:442–8. doi: 10.1097/SLA.0000000000005155 34387200 PMC8840990

[6] Noria SF Shelby RD Atkins KD Nguyen NT Gadde KM. Weight regain after bariatric surgery: scope of the problem, causes, prevention, and treatment. Curr Diab Rep. (2023) 23:31–42. doi: 10.1007/s11892-023-01498-z 36752995 PMC9906605

[7] Haughton SJ Gager SV Pinkney MP. Nutritional deficiencies following bariatric surgery: a rapid systematic review of case reports of vitamin and micronutrient deficiencies presenting more than two years post-surgery. Clin Obes. (2025) 15:e12658. doi: 10.1111/cob.70035 40719204 PMC12603345

[8] Miller-Matero LR Haley EN Loree AM Braciszewski JM Maye M Sehgal M . Post-surgical psychiatric symptoms, maladaptive eating patterns, and lifestyle behaviors associated with weight recurrence after bariatric surgery. Surg Obes Relat Dis. (2024) 20:297–303. doi: 10.1016/j.soard.2023.09.027 37923621 PMC11891465

[9] Wilding JPH Batterham RL Calanna S Davies M Van Gaal LF Lingvay I . Once-weekly semaglutide in adults with overweight or obesity. N Engl J Med. (2021) 384:989–1002. doi: 10.1056/NEJMoa2032183 33567185

